# Crystalline or Sponge Gold

**Published:** 1856-01

**Authors:** 


*Crystalline or Sponge Gold.*—It will be seen by a letter which we copy from a late No. of the New York Dental Recorder, that Messrs. A. J. Watts & Co., have recently made a most valuable and important improvement in the manufacture of this article. The senior editor has used some of the improved preparation, and he has no hesitation in pronouncing it vastly superior to any thing of the kind ever before employed by him for filling teeth. There was one objection to all the preparations of crystalline or sponge gold which he had previously used, and that is, its liability to crumble while introducing it into the cavity of a tooth, so that more or less of it escaped into the mouth, and was lost. This, however, while it is softer and more easily consolidated, is so adhesive, that considerable effort is required to separate a particle from the general mass. It is more easily moulded to the various depressions and irregularities of the surface of the walls of a cavity, than any previous preparation of the kind which we have seen. The particles or crystals are also more easily united one to another, and the union is less likely to be affected by moisture. From the experience which the senior editor has had in the use of the improved preparation, he feels warranted in saying, that a more perfect filling can be put in with it, in many cases, than with the best of foil, provided it be kept absolutely free from moisture during the process of introducing it into the tooth and compacting it. As much skill, however, and care are necessary in filling a tooth with this as with gold in any other form. In short, when properly worked, it cannot fail to give the most entire satisfaction.

